# When Antlers Grow Abnormally: A Hidden Disease Behind Common Cervid Trophy Deformities, Introducing Pedunculitis Chronica Deformans

**DOI:** 10.3390/ani15111530

**Published:** 2025-05-23

**Authors:** Farkas Sükösd, István Lakatos, Ádám Ürmös, Réka Karkas, Ákos Sükösd, Gábor Palánki, Attila Arany Tóth, Károly Erdélyi, Mihály Misó, Péter Gőbölös, Katalin Posta, Ferenc Kovács, Szilamér Ferenczi, Győző Horváth, László Szemethy, Zsuzsanna Szőke

**Affiliations:** 1 Department of Pathology, Albert Szent-Györgyi Medical School, University of Szeged, 6720 Szeged, Hungary; 2 Department of Regional Game Management, Ministry of Agriculture, 1052 Budapest, Hungary; istvan.lakatos@am.gov.hu (I.L.); ferenc.kovacs@am.gov.hu (F.K.); 3 Agrobiotechnology and Precision Breeding for Food Security National Laboratory, Institute of Genetics and Biotechnology, Department of Animal Biotechnology, Hungarian University of Agriculture and Life Sciences, 2100 Godollo, Hungary; ferenczine.szoke.zsuzsanna@uni-mate.hu; 4 Genome Integrity and DNA Repair Core Group, Hungarian Centre of Excellence for Molecular Medicine (HCEMM), 6728 Szeged, Hungary; adam.urmos@hcemm.eu; 5 Department of Oncotherapy, University of Szeged, 6720 Szeged, Hungary; karkas.reka@med.u-szeged.hu; 6 Department of Orthopaedics, Semmelweis University, 1083 Budapest, Hungary; sukosd.akos@semmelweis.hu; 7 Gyulaj Forestry & Hunting Plc., 7090 Tamasi, Hungary; palanki@gyulajzrt.hu (G.P.); titkarsag@gyulaj.hu (P.G.); 8 Department of Surgery, University of Veterinary Medicine, 1078 Budapest, Hungary; aaranytoth@yahoo.com; 9 HUN-REN Veterinary Medical Research Institute, 1143 Budapest, Hungary; erdelyi.karoly@vmri.hun-ren.hu; 10 Guth Forestry, NYÍRERDŐ Forestry Co., 4400 Nyiregyhaza, Hungary; mihalym825@gmail.com; 11 Department of Microbiology and Applied Biotechnology, Institute of Genetics and Biotechnology, Hungarian University of Agriculture and Life Sciences, 2100 Godollo, Hungary; posta.katalin@uni-mate.hu (K.P.); ferenczi.szilamer@koki.hun-ren.hu (S.F.); 12 Laboratory of Molecular Neuroendocrinology, Institute of Experimental Medicine, 1083 Budapest, Hungary; 13 Institute of Biology, Faculty of Sciences, University of Pécs, 7624 Pecs, Hungary; hgypte@gamma.ttk.pte.hu (G.H.); szemethy.laszlo@pte.hu (L.S.)

**Keywords:** fallow deer antler, antler abnormality, pathological fracture, Pedunculitis Chronica Deformans, cervids

## Abstract

For centuries, the most prevalent antler abnormalities observed worldwide have been attributed to trauma. However, detailed pathological investigation of these cases has not yet been carried out. In free-living fallow deer (*Dama dama*), we identified a chronic osteomyelitis-like condition—Pedunculitis Chronica Deformans (PCD)—using pathological and radiological diagnostics. We propose that inflammation during post-casting wound healing and consequent scar formation can trigger the development of PCD. In this study, we characterize the pathomorphology of PCD and introduce a scoring system to describe its severity. Furthermore, we describe the histoanatomy of the junction between the pedicle and the surrounding skin—an area essential for the integrity of the integument—which, when compromised, may predispose cervids to PCD. Our findings suggest that the most common antler abnormality results from a pathological fracture associated with PCD, which can be further complicated by fatal meningoencephalitis and brain abscesses. PCD-related lesions, while less frequently observed, can also be identified in roe deer (*Capreolus capreolus*) and red deer (*Cervus elaphus*), with species-specific differences. These findings overlap with cases reported in other cervid species, suggesting a more general disorder of antler formation. Describing this condition provides a basis for assessing its epidemiology and understanding its relevance to wildlife health.

## Simple Summary

Antlers are the fastest regenerating bone tissue in the animal kingdom and serve as biological superstructures reflecting genetics, nutrition, environmental conditions, and the quality of wildlife management. The most common forms of antler abnormalities have traditionally been attributed to injuries sustained during mating fights. However, their pathological background has not been systematically investigated. By examining the skulls, pedicles, and antlers of three deer species, we identified a pathological condition—Pedunculitis Chronica Deformans (PCD)—with features overlapping previously reported cases in other cervids. Resembling chronic osteomyelitis, PCD likely arises from inflammation during antler casting and regrowth. We present a novel scoring system to assess the severity of lesions and offer detailed anatomical and pathological characterizations to aid diagnosis. Our findings challenge the long-held assumption that most antler deformities are trauma-related and highlight the need for standardized recognition and deeper investigation into deer health and antler biology.

## 1. Introduction

Antlers are unique outgrowths that develop exclusively in males, with the exception of reindeer (*Rangifer tarandus*), specific to the Cervidae family that reflect the biological condition of the animal, exhibiting inherited phenotypes, nutritional status, environmental impacts, and the quality of wildlife management [1,2]. Notably, antlers represent the fastest-growing and regenerating bone tissue found in nature [1]. While antler abnormalities are rare [3], they hold significant value as trophies among hunters. The earliest scientific publication on antler deformities dates back to 1900 [4].

Rutting-related injuries and the resulting abnormal healing processes are currently regarded as the most widely accepted explanation for antler malformations [1,5,6,7,8]. The antler and pedicle together constitute a functional anatomical unit [9]. Consequently, it has been widely accepted that pedicle injuries are closely associated with antler deformities [6]. The factors that predispose this unit—primarily evolved for intraspecific combat [10,11]—to structural vulnerability remain poorly understood. Limb injuries have been reported to exert teratogenic effects on the contralateral antler, most likely via a complex and still obscure pathomechanism [1,5,6]. As secondary sexual characteristics, antlers are highly responsive to endocrine signals; in particular, reduced testosterone levels can induce distinct malformations such as “perruque-head”, “cactus buck”, or “antleroma”, which are generally distinguishable from other types of anomalies [12]. Moreover, nutritional factors, especially mineral intake, can significantly affect the integrity and quality of newly forming antlers [2].

Hungary is internationally recognized for the exceptional quality of its deer populations, with two world-record fallow deer (*Dama dama*) trophies originating from the northeastern region [13]. However, since the latter half of the 20th century, a notable increase in antler deformities has been observed in this species [14]. Similar abnormalities have also been observed in red deer (*Cervus elaphus*) and roe deer (*Capreolus capreolus*). Despite the significance of this issue, there are, unfortunately, no peer-reviewed English-language publications on the topic from Hungary or the broader Central European region. The only available source is a single case report published in the *Hungarian Veterinary Journal* over fifteen years ago, which did not include any epidemiological data [15]. In response, our research group was established as part of a government initiative to investigate and clarify the problem.

Additionally, such abnormalities have been noted among the elk (*Cervus canadensis*) population in Northern Arizona, including the Hualapai Indian Reservation [6]. Instances of comparable malformations were also documented on mule deer (*Odocoileus hemionus*) in central Utah in the mid-20th century [5], as well as in North-Central Kansas, where extreme antler malformations identical in morphology have been noted [12]. Particularly concerning are the distinctive osseous deformities involving the pedicle—the frontal bone outgrowth serving as the basis of the antler—and adjacent skull region. Such anomalies have been documented in white-tailed deer (*Odocoileus virginianus*) from Georgia, USA, where they were linked to peri-peduncular purulent inflammation and associated meningoencephalitis or intracranial abscess complications [16,17,18]. A comprehensive study conducted across 12 US states and four Canadian provinces found that 2.2% of 4500 white-tailed deer exhibited central nervous system inflammation coupled with pedicle, skull, and antler abnormalities [16]. This highly spatially variable disease was detected in 9 (35%) cases in a radio-collared male white-tailed deer population of 26 individuals aged 2.5 years or older in Kent County, Maryland, USA [19]. Despite these findings, the true extent and epidemiology of antler deformities remain unclear.

Without precise descriptions, it is not possible to distinguish between pathological abnormalities and physiological variants. In other words, proper treatment can only be carried out with the correct diagnosis. This approach allows the collection of a homogeneous group of diseased animals on which the causes can be properly investigated. Once identified, a treatment strategy can be developed. In addition, trophy evaluation procedures exclude cases showing such deformities, as the specimens concerned do not meet the criteria for standard evaluation https://cic-wildlife.org/about-the-tes-2/ (accessed on 18 May 2025). Consequently, the extent of these conditions remains underreported and poorly understood. Reliable epidemiological analysis requires a precise and consistently applicable phenotypic classification system, one that clearly distinguishes pathological changes from natural morphological variation and allows for straightforward subclassification, even in applied contexts such as trophy evaluation.

Our objective was to assemble a comprehensive collection of aberrant trophies from all three free-ranging cervid species in Hungary, focusing specifically on cases where the underlying cause could not be determined based on current scientific knowledge [1,5,6,7,8].

We hypothesized that the observable lesions could be graded by severity and, based on this assumption, developed a preliminary scoring system to characterize them. This system is intended as a foundation that can be refined through consensus-based classification using a larger sample set. In addition to macroscopic evaluation, we examined the surrounding soft tissues of both wet and dried antlers using histological and radiological methods, with the aim of identifying pathological patterns that might associate the lesions with known disease categories. We also sought to explore potential predisposing factors. Ultimately, if the constellation of findings supports the recognition of a distinct pathological entity, we intend to propose a formal nomenclature to ensure its clear and consistent identification in future research and evaluations.

## 2. Materials and Methods

### 2.1. Source of Anomalous and Normal Trophies

The study examined 50 *Dama dama* (DD) anomalous antler trophies collected between 2017 and 2021 from Hungary, Central Europe. A morphology control group (MCG) served as the basis of comparison to this evaluation, except for age, antler main beam length, and brow tine length. It comprised 24 trophies from Gúth, Hungary https://nyirerdo.hu/guthi-erdeszet/ (accessed on 18 May 2025), where no antler anomalies were reported during the same period. Trophies in both groups are from animals between 4 and 12 years old. Additionally, five cases of roe deer (*Capreolus capreolus*, CC) and three cases of red deer (*Cervus elaphus*, CE) were analyzed. During the course of the trophy collection, it became evident that DD was considerably more affected than CC or CE and exhibited a broader spectrum of lesions compared to the latter two species. The observed overlap in the nature of distortions across species suggested that a common underlying process might be manifesting in species-specific ways. Consequently, we opted for a separate evaluation by species, applying the same diagnostic criteria. Given the notable differences in case numbers, we also employed distinct statistical approaches for each species. This strategy aimed to determine whether the phenomenon was species-specific or indicative of a broader condition affecting cervids in general.

The trophy-based age estimation was performed following established methods described in the literature [20,21]. The classic CIC (International Council for Game and Wildlife Conservation) trophy evaluation includes the assessment of age, antler beam length, and brow tine length measurements; therefore, we could use these data from the Hungarian Trophy Register (HTR) [22]. From this, a trophy register control group (TRCG) was formed from 2912 antler evaluation sheets, excluding those marked as “deformed”, “injured pedicle”, or “diseased pedicle”.

### 2.2. Macroscopic Examinations

A standardized protocol was developed for the macroscopic evaluation. The morphological deviation of the trophy from the normal was evaluated for the roses (R), antlers (A), pedicle (P) and skull (S) with 3, 6, 7, and 6 parameters, respectively, for a total of 22 parameters on both sides in 24 healthy (MCG) and 50 abnormal cases (Table 1). An experienced wildlife manager (IL) with decades of trophy assessment experience and a consultant surgical pathologist (FS) conducted the initial assessments. To ensure inter-observer reliability, two additional trained observers (R.K. and A.S.) independently evaluated a subset of trophies.

Furthermore, the RAPS features were sorted into 4 severity grades based on their pathomorphological background (Table 2).

We also analyzed a left abnormal antler in the velvet phase that was found incidentally within our study area. Above the frontal tine, an additional, aberrant branch of nearly equal length was observed. Near the site corresponding to the expected ablation surface, a highly irregular plane covered with blood coagulum was visible. The velvet skin showed no epithelial defects, fractures, or any abnormalities indicative of mechanical trauma.

### 2.3. Analysis of Pedicle Ellipticity

We tested whether PCD induces elliptical distortion of the pedicle using the minor (*d*) and major (*D*) axes of pedicles (modelled as ellipses). Ellipticity was quantified using the distance of the foci from the origin ($\sqrt{(D/2{)}^{2}-(d/2{)}^{2}}$) and eccentricity (foci distance/D).

The diameters *D* and *d* were measured on both sides at the highest measurable plane of the pedicle in 33 healthy and 14 abnormal fallow deer (*Dama dama*) trophies using a caliper (see Table S5). In the healthy group, the average values for *D* and *d* were 44 mm and 41 mm, respectively, with an average calculated difference of 3.12 mm. In the abnormal group, the mean *D* was 64.4 mm and *d* was 47.4 mm, resulting in an average difference of 10.78 mm.

Both shape descriptors, ellipticity and eccentricity, showed statistically significant differences between the healthy and abnormal groups, as determined by two-sample Wilcoxon tests (*p*-values: 4.371 × 10^−7^ and 1.426 × 10^−4^, respectively).

### 2.4. Microscopic Examinations

Five DD heads, each showing abnormality, both in the velvet phase and with fully ossified (dry) antlers, were collected. All specimens exhibited evident abnormalities on at least one antler side and included soft tissues such as skin, connective tissue, muscle, and brain. The proximal ca. 20 cm long segment of the antler, the whole antler in case of obvious abnormality, and the pedicle attached to the antler and the surrounding skull roof, including all soft tissues and brain, were cut out and fixed in 10% formalin for at least one week. These preserves were sawed into 4 mm thick slices with a meat industry band saw. Visible alterations were excised for microscopic examination. Electric decalcification using a Tissue-tek TDE 30 Decalcifier system (ref.: 1428 Sakura Finetek Europe, Alphen aan den Rijn, The Netherlands) was used for three weeks with continuous monitoring. Samples that couldn’t be sectioned after this time were further handled for one month in a solution of 70% ethanol containing 5% phenol or Q path DC3 (VWR ref.: 09128300. Vienna, Austria) for three weeks.

After decalcification, traditional pathological techniques (embedding in a paraffin block and making 5 micrometer sections) were employed, including Hematoxylin & Eosin, PAS, Warthin-Starry, Giemsa, Zhiel-Neelsen, and Wade-Fite staining to detect potential pathogens. In four cases of yearling stag trophies, parallel 4 mm thick slices were sawed from the pedicle, and after decalcification, microscopic examinations were conducted.

### 2.5. Radiological Examinations

Computed tomography (CT) images of 16 deer trophies and 2 complete heads with soft tissue were acquired with a single slice GE CT Scanner Model CT/e (GE Healthcare, Chicago, IL, USA). Acquisition parameters for CT images were as follows: helical mode, 512 × 512 matrix, 1 mm slice thickness, 1 mm image interval, 120 kVp, 20–120 mA, and bone convolution kernel. The preparations were placed in a prone position on the CT bed with the aid of foam wedges. Transverse images of the skull were obtained perpendicular to the hard palate. The scanned area ranged from the occipital bone to the mid-orbital region, including the antler tree. The osseous structures on DICOM images were evaluated using a window width of 4000 and a level of 400 HU.

### 2.6. Statistical Analysis

The presence of morphological anomalies (R, A, P, and S) was recorded with a value of 1 for presence and 0 for absence, separately for each side. A2.1 and A3.1 were considered as present if the brow tine and main beam length were lower than the bottom 20% and 5% quantiles of the distributions of brow tine and main beam length in the TRCG of the corresponding age and side. Pairwise comparisons between means, unless stated otherwise, were carried out using two-sample Wilcoxon tests, with Holm’s multiple testing correction. Computations were performed using the R language and environment version 4.4.3 [23], figures were drawn using ggplot2 [24], ggpubr [25], ggraph [26], and pheatmap [27].

## 3. Results and Discussion

### 3.1. Epidemiology of Cervidae with Aberrant Trophies

**Figure 1:**
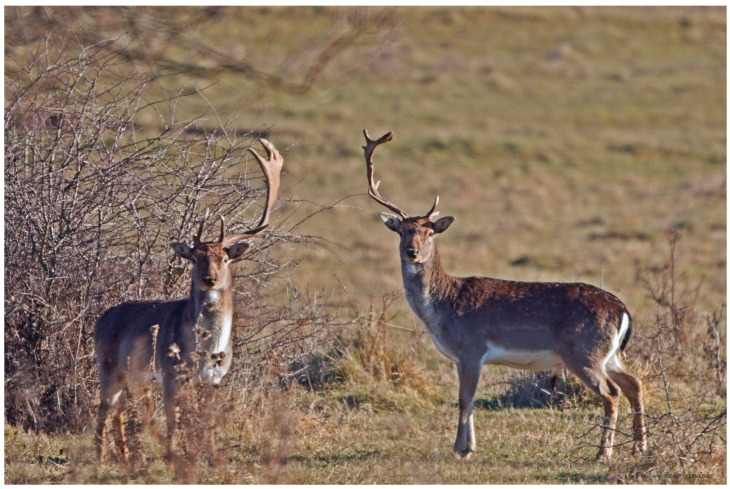
A striking scene from Kocsola (Southern Hungary, Central Europe) showing three fallow deer (*Dama dama*) with antler development issues. Their upright head posture suggests that their antlers did not break off during fights but failed to develop properly. To compensate for the resulting weight imbalance, they developed hypertrophied neck muscles to maintain normal head position and vision (Photo: András Molnár).

The analysis of our Southern Hungarian non-species-selected aberrant trophy collection from 2017 to 2021 showed significant occurrences in antler anomalies in various free-living Cervidae species. The incidence of aberrant trophies among fallow deer (*Dama dama*, DD) was notably higher than in roe deer (*Capreolus capreolus*, CC) and red deer (*Cervus elaphus*, CE), with ten times more anomalies in DD compared to CC (50 vs. 5) and approximately 16 times more than CE (50 vs. 3). When adjusted for population density, DD appears to be impacted the most by these deformities: 34 times more frequently than CC and 50 times more than CE, suggesting a unique vulnerability (Figure 1, Table S1).

### 3.2. Health Condition of Fallow Deer (Dama dama) with Aberrant Trophies

**Figure 2:**
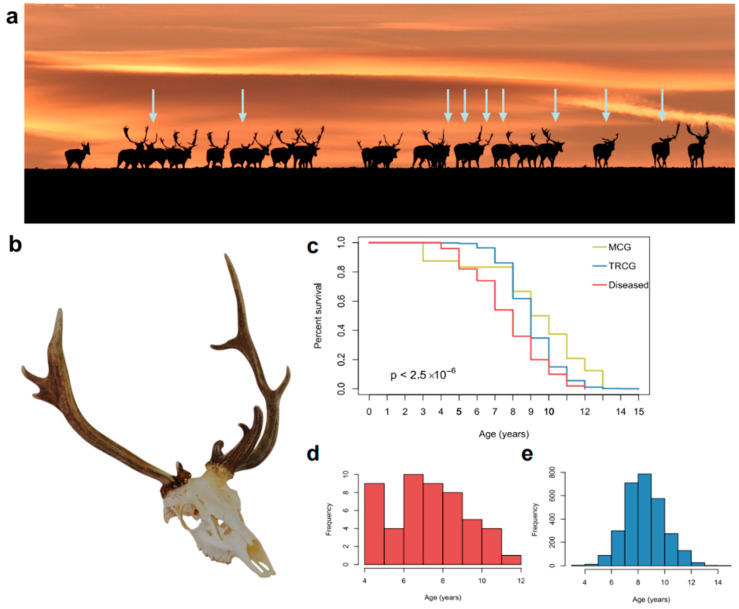
Antler abnormality in Southern Hungary. (**a**) A herd of fallow deer *(Dama dama*, DD) at sunset in the Kocsola region (Southern Hungary). Notably, 9 out of 23 individuals (39%) exhibit visible antler abnormalities (arrows) (Photo: Sándor Juhos). (**b**) Individuals with antler anomalies, like those shown in the photograph, are excluded from trophy evaluations, making it difficult to obtain reliable data on their prevalence. (**c**) Although hunting-related mortality is influenced by planned culling, it is still associated with the animal’s health status. Kaplan-Meier survival analysis revealed reduced life expectancy for animals with deformed antlers compared with healthy members of Morphological (MCG, *n* = 50) and Trophy Register (TRCG, *n* = 2912) Control Group (Kaplan-Meier curves contrasted with log-rank test). (**d**) The average age of the healthy (not showing antler abnormality) individuals to be evaluated is 9 years. (**e**) Compared to an average age of 7.74 years for aberrant DD trophies (*p* < 0.0001).

In the Hungarian trophy register (HTR), data on age, main beam, and brow tine sizes are recorded for normal trophies from 2017 to 2020; a total of 2924 register sheets documented these parameters regarding our study region. Of these, 12 cases (0.4%) included pedicle abnormalities and were excluded from analysis. The remaining 2912 records formed our trophy register control group for brow tine and main beam sizes across age groups. This low incidence rate in records contrasts sharply with the 40–80% frequency of abnormalities observed during harvests in this region [14] (Figure 2a). The discrepancy may be due to the lack of standardized methods for describing malformed trophies. Scoring systems such as those by the International Council for Game and Wildlife Conservation (CIC score, https://www.cic-wildlife.org (accessed on 18 May 2025)) and Boone and Crockett Club (B&C score, https://www.boone-crockett.org (accessed on 18 May 2025)) are well-suited for assessing healthy trophy sizes and forms but lack guidelines for classifying malformations (Figure 2b). Consequently, only sporadic notes on these anomalies appear in trophy evaluations. The remaining 2900 healthy cases formed our trophy register control group (TRCG), with an average age of 9 years, compared to an average age of 7.74 years for aberrant DD trophies (*p* < 0.0001) (Figure 2c,d).

### 3.3. Characteristics of Fallow Deer (Dama dama) Aberrant Trophies

**Figure 3:**
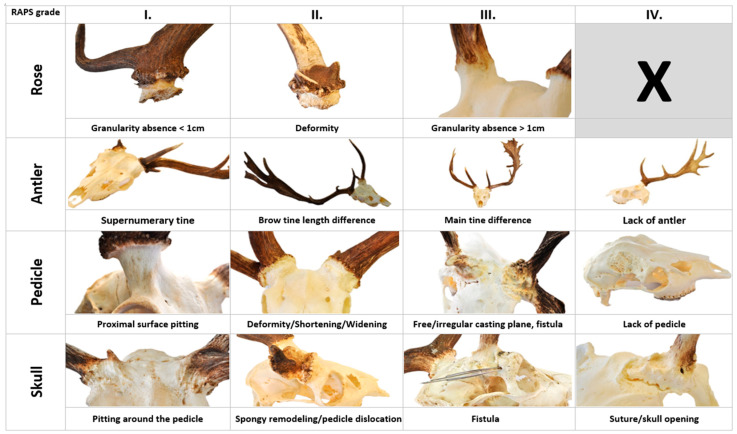
The assessment of the RAPS (rose/pedicle/antler/skull) features describes Pedunculitis Chronica Deformans (PCD) and its severity. The RAPS features can be grouped into three grades for the rose and four for the pedicle/antler/skull. **Grade I:** Lack of continuity in the grain of the rose is longer than 5 mm but less than 1 cm. (**R1**). A supernumerary tine originating from the rose can be a sign of an extra antler bud (**A1**). Pitting on the proximal surface of the pedicle (**P1**). The pitting extends to the skull surface (**S1**). **Grade II:** A deformity of the rose that is visible to the eye is a deviation from a regular circle. This is not independent of the pedicle deformity (**P2**) and visible on cast antlers, too (**R2**). The length difference between the two sides of the brow tines (**A2**) and the difference from the trophy register control group (**A2.1**), which is derived from data of the Hungarian trophy register after exclusion of cases marked abnormal (see text). The pedicle deformity characteristic of PCD in fallow deer (*Dama dama*) is a difference in the major and minor axis lengths of more than 4 mm, also visible by eye (**P2**). Shortening (**P3**) and widening (**P4**) of the pedicle beyond normal extent. Spongy remodeling of skull bones where the cavities are larger than at intertrabecular spaces in cancellous bone (**S2**) or visible in the center of the pedicle has shifted significantly, mostly towards the brow ridge (**S6**). **Grade III:** Loss of rose granularity is greater than 1 cm (**R3**). The difference in length of the main beams relative to each other (**A3**) and the difference relative to the Trophy Register Control group (**A3.1**). A pedicle fistula (**P5**, or a free casting plane without any protruding antler-bony structure (**P6**). A fistula mouth opens distant from the pedicle on the skull (**S4**). **Grade IV:** Absence of antlers or aberrant antler beams (**A4**). More than fifty percent of the casting plane of the pedicle is in or below the plane of the skull, which is considered to be the absence of it (**P7**). Discohesion along the interosseous suture (**S3**) or separation of cortical bone independently is a sign of bone displacement (**S4**).

Aberrant trophies exhibit pathological variations that are not observed under physiological conditions, for example, significant absence of rose granularity, remarkable differences between beam lengths, pedicle deformities, and skull abnormalities. Additionally, they often show the accumulation of unique anatomical variations rarely observed under normal circumstances, such as supernumerary tines. To characterize these abnormalities, which we dubbed RAPS features, we examined three malformations of the antler rose (R1–R3), six of the antler (A1, A2, A2.1, A3, A3.1, and A4), seven of the pedicle (P1–P7), and six of the surrounding skull (S1–S6), altogether 22 characteristics (Table 1). Since specific combinations of RAPS symptoms can also indicate disease severity, we established four grades of disease progression (Table 2 and Table S4, Figure 3) based on this framework, which can serve as a starting point for a consensus classification based on a larger cohort in the future.

**Figure 4:**
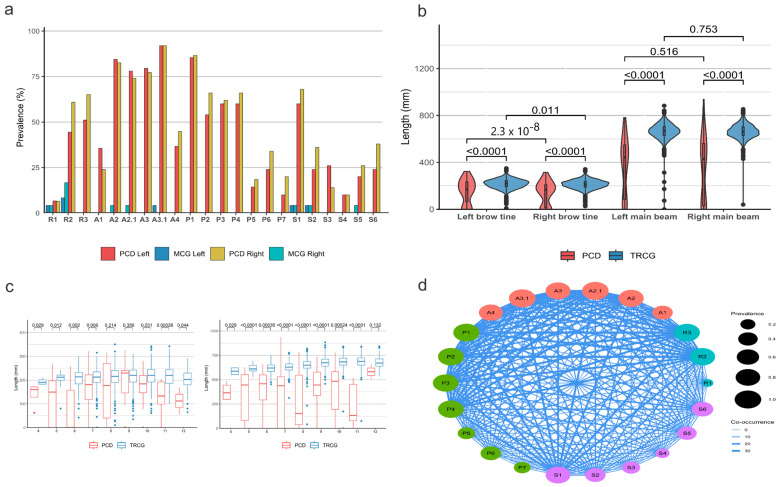
Prevalence of RAPS features representing anomalies of the rose (R), antler (A), pedicle (P), and skull (S) in fallow deer (*Dama dama*). (**a**) Fifty trophies with antler abnormalities (left side: red and right side: yellow) and 24 trophies from a healthy (morphological) control group (left side: blue and right side: teal) were evaluated. Some anomalies were observed in the healthy cohort, but at low frequency (Table S4). (**b**) Length differences between anomalous DD (red) and healthy (blue) brow tines and main beams by side in comparison to the trophy register control group (TRCG, *n* = 2912). Numbers represent *p*-values of two-sample Wilcoxon tests, with Holm’s multiple testing correction. Differences between sides were non-significant in all but one of the comparisons (TRCG brow tine), reflecting the notion that such differences often arise as a result of normal physiology. (**c**) Differences in brow tine and main beam lengths between abnormal DD individuals and the TRCG by age group. Numbers represent *p*-values of two-sample Wilcoxon tests, with Holm’s correction. Brow tine lengths were significantly different in most, and main beam lengths in all but one age of the groups. (**d**) The graph shows the co-occurrence of the RAPS symptom in the 50 anomalous DD trophies. The size of the nodes represents the prevalence of the symptom in the cohort, and the width of the edges represents the number of co-occurrences between the two symptoms. The data were aggregated by side (see also Figure S1).

We compared the frequency of RAPS features between a healthy morphological control group (MCG) and aberrant-antlered DD cohorts. On the MCG (24 trophies) from the control, we inspected 1056 morphological features, of which 16 (1.52%) showed anomalies. In contrast, among aberrant DD trophies (50 trophies, 2200 inspected features), 989 pathological features were detected (44.95%) (Table S4). The Fisher’s exact test (*p* < 0.0001) confirmed that the RAPS characteristics were highly discriminative between these groups (Figure 4a). All of the RAPS features showed a consistently higher prevalence in the aberrant trophies than in the MCG, with 20 of the 22 being statistically significant discriminators (Pearson’s Chi-square tests, see Table S4).

While antler abnormalities are generally considered unilateral lesions, our study suggests a bilateral condition (Table S4). The noticeable asymmetry likely reflects varying environmental factors, primarily infections and secondary traumas, as previously suggested [28]. This chronic process, which can span several years and multiple antler shedding cycles, becomes more pronounced on the side that is more severely affected.

In our study, the most frequently observed anomaly was compared to the TRCG—a reduction in antler main beam length (A3.1, 92%) (Figure 4b,c). Additionally, visible asymmetry between the main beam length was frequently noted (A3, 69%).

**Figure 5:**
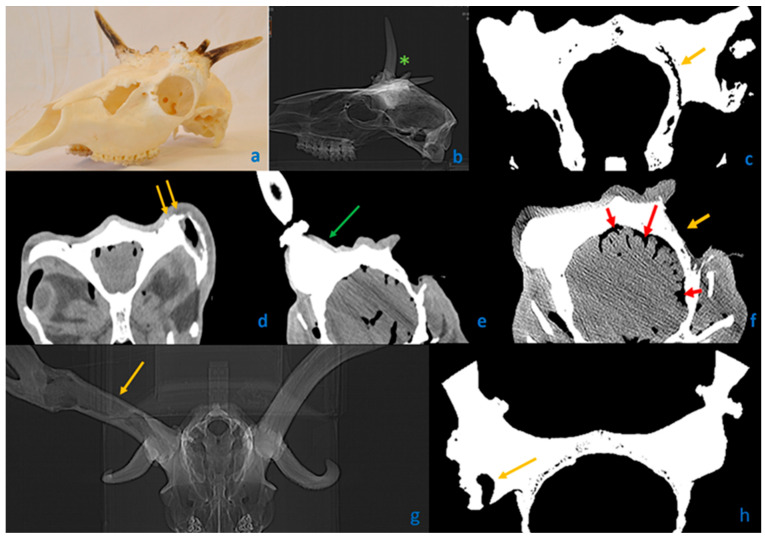
Computed tomography (CT) scans of three fallow deer (*Dama dama*) cases. (**a**–**c**) A trophy with severe involvement on both sides with aberrant antler beams (AAB). (**d**,**e**) Complete head with soft tissues. (**g**,**h**) The only case with cavity formation in an antler. (**a**) Bilateral loss of antlers with deformed pedicles and AAB. (**b**) Typically, no cavities are formed in AABs (marked with green asterisks). (**c**) Frontal scan through the antler pedicles. On the left side, not visible to the naked eye, a large gap-like cavity (yellow arrow) ran parallel to the cranial plane without displacement of the cranial bones. Development due to trauma is unlikely. (**d**) Whole head with soft tissues. Yellow arrows show the unevenness of the residual pedicle, suggestive of superficial chronic osteomyelitis. (**e**) The green arrow shows the deeper plane of the other side pedicle surface without significant radiographic abnormality. (**f**) In an even deeper plane, due to pus accumulation in the subcranial space, the widening of the sulci and thinning of the gyri (red arrows) suggest meningoencephalitis. The yellow arrow shows the lack of a pedicle. (**g**) Only one of our cases showed a cavity (yellow arrow) in the unilateral antler beam, which may indicate the death of the growth center cells during growth. (**h**) Necrotic cells may have exited through a fistula (yellow arrow) to the outside.

The second most frequent change was the pedicle surface pitting (P.1, 80%), particularly visible on the frontal area, described earlier by Davidson et al. as a hallmark of this anomaly [29]. Histological and radiological analyses revealed that this accompanies the superficial form of chronic osteomyelitis [30,31,32] (Figure 5d,e).

This often extended to the adjacent skull (S1, 64%). The rose deformity (R2, 48%) and discontinuity of rose granularity greater than 1 cm (R3, 53%) were also prevalent, appearing in roughly half of the cases. A prior study has noted the association of R2 with unilateral antler deficiencies [28]. In our study, R2 was significantly associated with visible brow tine difference (A2, 76%), while R3 correlated with shortened brow tine (A2.1, 76%) and main beam (A3.1) length. Furthermore, both R2 and R3 were significantly associated with all pedicle and skull anomalies (Figure 4c and Table S4), indicating that the more severe rose abnormalities can act as an indicator of subcutaneous issues. The irregular, also called “dirty” casting plane (P6, 29%) in shed antlers is a well-documented phenomenon [1], associated with an insufficient testosterone peak [33]. However, only a third (29%) of the trophies displayed this characteristic, as many were collected outside the casting period. The shortening and widening of the pedicle is a physiological event that occurs with age [1,34]. Based on subjective judgment, shortening (P3) was considered more pronounced than normal in 61% of abnormal cases and widening (P4) in 63%. However, the pedicle deformity (P2), defined as a deviation from a regular circle shape, appeared in over half of our cases (60%), serving as a usable marker of disease recognition in DD but not in CC and CE. On average, the larger diameter of the near-circular cross-section in healthy antlers exceeded the smaller diameter by only 3.12 mm. In contrast, antlers from abnormal trophies showed a threefold greater difference, averaging 10.78 mm (Table S5). Both elliptical shape descriptors—ellipticity and eccentricity—differed significantly between the healthy and abnormal groups (*p*-values: 4.371 × 10^−7^ and 1.426 × 10^−4^, respectively). To the best of our knowledge, these are the first results evaluating pedicle ellipticity, based on which we have proposed a 4 mm threshold between the diameters *D* and *d*. According to this criterion, 13 out of 66 antlers (19%) from the normal group fell within the abnormal range, while 3 out of 28 antlers (10%) from the PCD group did not exceed the threshold (Table S5). More precise conclusions will require further studies with larger sample sizes.

In extreme cases, lateral displacement of the remaining pedicle, often extending to the supraorbital ridge (S6, 31%) was observed, similarly to the phenomenon photographically documented in North Arizona elk (*Cervus canadensis*) populations by J.L. Rachlow [6]. These aberrant proliferation–remodelling features (P2 and S6) are similar to those described in our study, suggesting that it is not just a DD-specific phenomenon.

In our work, we defined “spikes” (a term used within the hunting community) as aberrant antler beams (AAB) to distinguish terminologically between diseased forms and healthy yearling stags without branched antlers. If nothing or only an AAB was present at a given site, we classified the specimen as “lacking antler” (A4, 40%). We considered a formation as an antler if the main beam exceeded the brow tine in length, with at least a rudimentary antler rose. CT scans revealed these structures to be generally solid, with only one case showing cavity formation (Figure 5g,h). In cases where pedicle blastemas are less involved, more pronounced growth can occur by incorporating unused nutrients and minerals, which become more available due to a reduced number of osteoblasts in other areas. We termed this process compensatory growth (CG). The effect might be analogous to contralateral antler growth following removal of the peri-peduncular periosteum described by Li et al. [35]. CG can be indicated by a significant difference in brow tine length due to a longer-than-normal brow tine on the healthy side.

**Figure 6:**
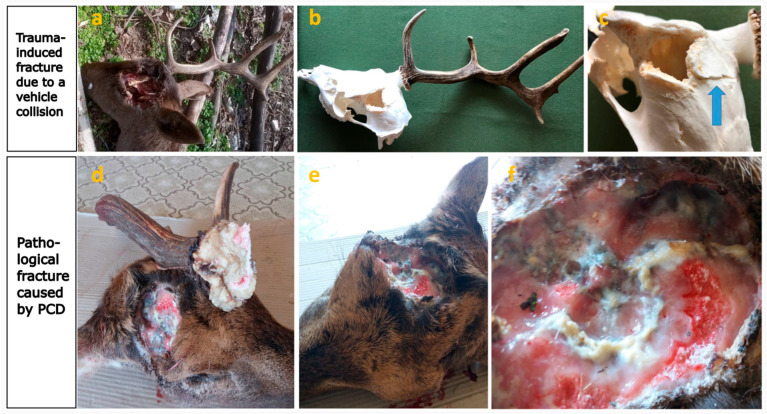
The difference between an obvious trauma-induced and a pathological fracture. (**a**) The top row of images shows the left antler broken out following a motor vehicle collision. (**b**) Condition after soft tissue removal. (**c**) The fracture surfaces were sharp and angular, with displacement of the cranial bone (arrow). (**d**) The bottom row shows the right antler and the site of the breakout, which occurred due to a fall following a hunter’s gunshot. Under normal circumstances, a fracture should not occur in this case. However, an underlying disease, secondarily infected with pyogenic bacteria, weakened the bone structure to such an extent that it fractured even under minimal force. The surface of the breakout, which covered a large area, was rounded. (**e**) Loss of the left antler occurred earlier. (**f**) Most of the fracture surfaces were covered with greyish-white pus, indicating a superinfection with pyogenic bacteria.

Among the observed skull abnormalities, irregularities such as peri-peduncular skull surface pitting [29] (S1, 64%), spongious skull remodeling (S2, 30%), and the lateral displacement of the expanding pedicle (S6) suggest atypical resorption and proliferative processes cannot be solely attributed to trauma or displacement during healing. A morphological evidence of secondary healing of chronic osteomyelitis is fistula formation [30,36], which was detected on the pedicle (P5) in 16% of the cases and on the skull (S4) in 10% of the cases. The opening of sutures (S3, 20%), theoretically, might be a consequence of injury; however, it would not occur in isolation since it typically requires adjacent skull fractures and dislocations. Trauma-induced extensive detachment of the external cortical plate from the underlying bone layer would result in substantial displacement. Conversely, CT imaging (Figure 5c) of an aberrant trophy revealed that the gaps between cortical plates (S5, 23%) often aligned with the cranial contour, suggesting that the origin of this malformation is unrelated to trauma. The absence of any signs of sharp fracture lines—either with or without displacement—and the lack of callus formation or (at least) partially broken pedicle structures (f.e., regular circular cross-section, with a missing part) further support that these abnormalities are not typical traumatic injuries. If not only trophies are available but also soft tissues can be examined, the presence of abscess-like purulent inflammation is evidence of the underlying pathology (Figure 6d–f).

**Figure 7:**
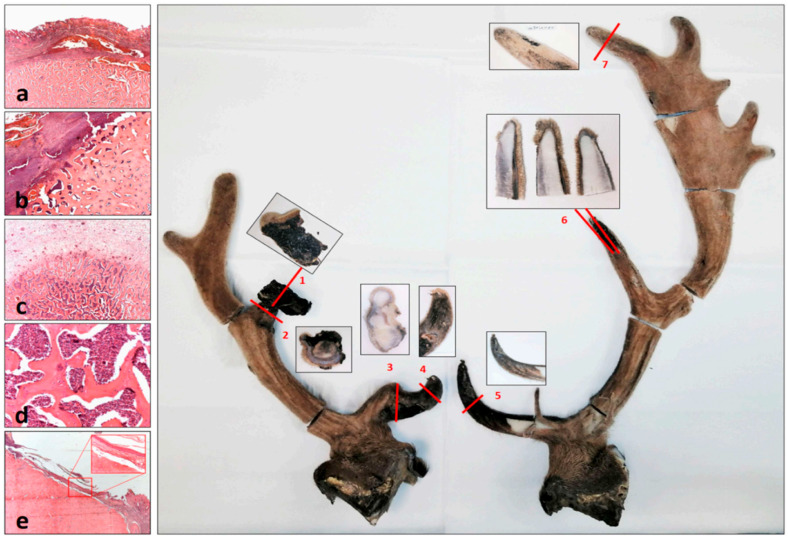
Microscopic examination of wounds caused by injury in the velvet phase, leading to antler deformity and asymmetry, suggests acute inflammation. The significant difference between the sides may be related to the different extent of the injuries and subsequent inflammation, and, therefore, a difference in the duration of the recovery period under which the antler could grow. The red numbers show the directions of the cut-offs for microscopic examination, and the adjacent images show the cut surfaces of the planes. (**a**) Subcutaneous hemorrhage and detachment from the bone surface are indications of injury (Cut 7). (**b**) The larger lesion was associated with necrosis of the skin and periosteum (upper left half of the figure). The superficially infiltrated blood layer confirmed that this was caused by trauma (Cut 2). (**c**) Inflammatory cells also reached the deep cancellous bone tissue (Cut 5). (**d**) The intertrabecular spaces of the cancellous bone were filled with acute inflammatory cells (neutrophil granulocytes) (Cut 5). (**e**) During re-epithelialization, the epithelium slides from the skin surface adjacent to the lesion to the free bone surface. The resulting contact is consistent with one of the histological components of the peduncular–dermal junction (PDJ) and is indicative of a longer period of regeneration. The inset shows a bony–epithelial contact area without the dermis. The gap is a retraction artifact formed during fixation (Cut 6).

The prevailing hypothesis suggests that post-traumatic deformity within antler pedicles is a consequence of the misalignment of the broken surfaces since the periosteum and skin cannot hold them together properly [1,28]. Certain studies seemingly supported this theory by examining the mechanical disruption of the pedicle immediately after casting or of antlers during the velvet stage [1]. However, it is important to note that these experiments were limited to growing antlers, which may represent imperfect antler growth rather than the process of pathological healing after the breakout of dry antlers [37,38]. In our study, we examined one case where injury occurred during the velvet stage, which caused acute osteomyelitis, accompanied by re-epithelialization of the exposed velvet surface (Figure 7). This inflammation was localized within the injured area and its immediate vicinity, sparing the deeper marrow area in larger tines and avoiding extension to the pedicle. Consequently, healing produced a deformed antler with a size reduction proportional to the initial injury. In theory, a severe acute inflammation that reaches the pedicle could cause a fatal sepsis. A less severe process, confined to the antlers, can cause deformation or breakage of branches [28,39]. However, animals mostly avoid injury during the velvet stage due to the innervated and sensitive nature of their antlers [1,28]. In the absence of chronic inflammation or bone remodeling, injuries at the velvet stage are not usually a significant cause of antler deformities; healthy antlers can regrow in the following year [1]. Our biobank reflects only one such case among other abnormal samples.

### 3.4. Antler Anomalies in Roe Deer (Capreolus capreolus) Trophies

Our aberrant Cervidae trophy collection included five roe deer (*Capreolus capreolus*, CC) trophies exhibiting antler anomalies overlapping with symptoms of DD. The antlered population-based data [22] indicate that this species was affected 22 times less frequently than DD. However, we believe this observation does not accurately reflect the true prevalence of antler anomalies in CC populations. This is because normal trophies exhibit significant morphological variability, which can make it difficult to recognize aberrant ones. Additionally, obvious anomalies may lead to exclusion from the evaluation process (https://www.cicukteb.com/uk-species/index (accessed on 18 May 2025)).

**Figure 8:**
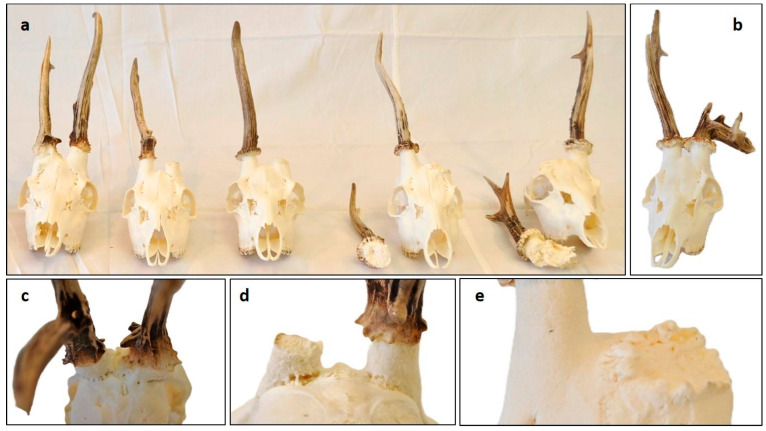
Pedunculitis Chronica Deformans (PCD) caused trophy anomalies on roe deer (*Capreolus capreolus*, CC). (**a**) Our five cases, ordered by severity, showed signs of PCD. (**b**) One velvet-phase injured CC with intact rose, pedicle, and skull; therefore, the deformity in the antlers is not due to PCD. (**c**) CC Case 1, as seen from behind. There are no brow tines or antler burrs on either side. (**d**) CC case 2 seen from behind. The distal pedicle surface showed pitting on both sides, more prominently on the side from which premature casting occurred. On this side, fissure formation between the pedicle and frontal bone and on the other side, the loss of burr granulation are evident. (**e**) On the left side of CC case 4, a margin crest developed on the broadening pedicle base, showing osteoblastic bone building activity.

Due to the limited number of cases, we categorized and analyzed the involvement of the rose (R), antler (A), pedicle (P), and skull (S) together. We assessed the RAPS features on five trophies on both sides and categorized the frequency of anomalies into three groups: Frequent (70–100%), moderately common (40–69%), rare (less than 39%) (Figure 8, Table S2) In two cases (CC4 and CC5), one of the antlers broke off during the hunt, indicating significant weakening of the bony structure that led to pathological fractures. In these cases, we were able to evaluate the broken part and compare it to the intact side. Overall, antler anomalies were the most frequent alterations (63%), occurring more often than pedicle (27%) and skull (23%) anomalies. Interestingly, the incidence rate of rose anomalies (R1–R3) was much lower (21%) than in DD and CE. This could be a consequence of the absence of pedicle deformation (P2), which is common in DD. Since R2 is not independent of P2, this may explain the interspecies difference.

This difference could be attributed to variations in osteoclast distribution in the pedicle. In CC, osteoclasts are concentrated narrowly around the casting plane, leading to a nearly flat shedding plane upon activation. In contrast, in DD, osteoclast distribution is more dispersed along the pedicle, resulting in a more uneven casting plane.

### 3.5. Occurrence of RAPS Features in Red Deer (Cervus elaphus)

**Figure 9:**
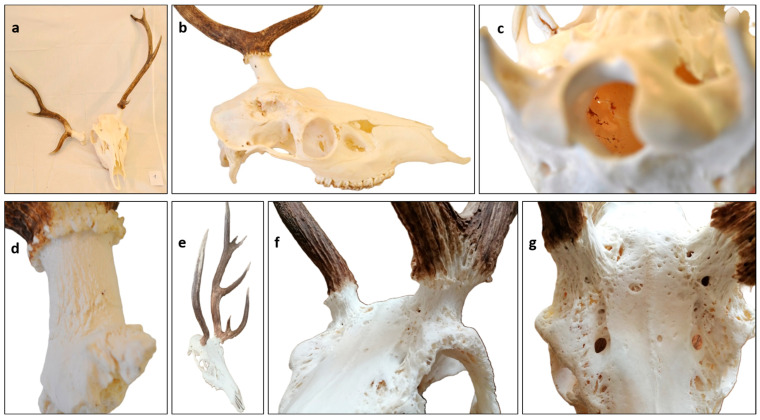
Signs of Pedunculitis Chronica Deformans (PCD) on red deer (*Cervus elaphus*, CE). (**a**–**d**) A two-year-old stag trophy with a broken-out right pedicle. The fracture happened during the hunt, therefore, we could examine both sides. (**a**) The fracture pattern serves as an example of juvenile PCD, where aseptic bone necrosis leads to pathological fracture. The right mean beam was shorter and less developed than the left. (**b**) The broad breaking surface involved most of the frontal bone and approached the orbital arch. (**c**) View through the foramen magnum towards the right pedicle base. The spongy perforations occurred without displacement of the skull bone surface and were not situated along a line of force, which could be a sign of a traumatic impact. (**d**) The broken-out pedicle showed remarkable pitting on the proximal two-thirds of its surface without deformation or loss of rose granulation. (**e**–**g**) A case of a five-year-old stag as an example where the inhibited cast wound healing could lead to an adult-type PCD. (**e**) The difference between the two sides was prominent, as only an aberrant antler beam developed on the right side. (There is no brow tine; therefore, we cannot consider it as an antler). (**f**) Total loss of rose granularity on both sides without pedicle shortening, broadening, or deformation, but surface irregularity is obvious. (**g**) The spongy bone remodeling due to osteoclast activation is a hallmark of PCD in CE. On both sides, more of the frontal bones were involved, and the disease was reaching the orbital arches too. In contrast to fallow deer (*Dama dama*), there is no bony crest formation and broadening of the pedicle, which could be a sign of elevated osteoblast activity.

The antlered population density [22] adjusted data referred to 32 times less involvement in red deer (*Cervus elaphus*, CE) than fallow deer (*Dama dama*, DD) (Table S1). Despite the accessible low number of CE cases, RAPS features showed a significant overlap between these two species (Figure 9 and Table S3). A conspicuous weakening by abnormal remodeling of the pedicle at least on one side was visible in those CE cases. In one three-year-old stag case (CE 1), the right pedicle broke out (P6, P7) during the hunt. The fracture happened on the most proximal third of the pedicle immediately at the level of the skull, which showed spongious reconstruction (S2) of the outer and inner surfaces without dislocation of the flat bone lamels (Figure 9b,c). Further on, the holes in the inner surface of the pedicle did not arrange along a fault line, suggesting that this relatively large loss of bony material could not have been a consequence of trauma (Figure 9c). The pitting on the outer skull surface (S1) around the pedicle is a clear sign of chronic osteomyelitis (Figure 9d), which is a result of long-lasting inflammation, not acute trauma. The morphological evidence of this pathomechanism includes pitting of the distal part of the pedicle (P1) and skull surface (S1), or in serious cases, the spongious remodeling of this flat bone (S2). The improper wound healing with scar formation can lead to bone weakening, which can elicit distal pedicle, rose, and antler deformation or even loss (Figure 9e). One of the most conspicuous differences between CE and DD was the extent of spongious remodeling (Figure 9f). This was not only more prominent in CE, but involved the frontal bones extensively, even reaching the sutures in some cases (Figure 9g). This could be the consequence of the osteoclastic/osteoblastic activity (which is normally most prominent on the casting plane), not limited to pedicles, but involving the surrounding part of the frontal bone. Additional features different from DD are the lack of deformation (P2), prominent shortening (P3), and widening (P4) of the pedicle, which can be explained by interspecies differences. In the case of DD, during bone remodeling by chronic osteomyelitis, the balance between osteolytic osteoclast activity and the proliferation activity of osteoblasts shifts in favor of the latter, as opposed to CE where the former can be more prominent.

### 3.6. Considerations for RAPS Score Assessment

Despite their rigorous applicability, the RAPS features cannot be treated as entirely independent variables due to inherent conflicts and contingencies. For instance, R1 and R2 are mutually exclusive events. Additionally, A2 and A3 were determined by comparing the two sides, and to maintain consistency, we assigned a score of 1 on both sides if present. However, this approach introduces an inherent bias into the downstream analysis by affecting the evaluation of laterality. Furthermore, our observations indicated that the age-stratified brow tine and main beam length distributions in the trophy register control group (TRCG) were often skewed to the left. As a result, for the assessment of A2.1, we implemented a less stringent bottom 20% threshold, which allowed for a greater than 15% increase in sensitivity for this marker.

### 3.7. Healing of the Casting Wound

Upon casting, a wound corresponding to an open bone fracture forms at the detachment site. In healthy animals, this wound generally only leaves at most a small central scar after healing, and eventually is enclosed in velvet [40].

Wound healing is a complex and delicate process that can sometimes falter, leading to chronic, non-healing wounds. Common initiating factors in such cases include infection, persistent inflammation, immunosuppression, and impaired circulation, often working together to hinder recovery [40,41]. Despite the regenerative potential observed in antler wounds, research has not yet focused on the presence of inflammatory cells—essential indicators of immune activity—or on the morphological traits associated with pathological healing in antlers. In a non-sterile environment, wounds are susceptible to microbial colonization. This poses a risk to the ossification zone as bacteria can spread through the bloodstream, potentially causing osteomyelitis [28]. However, when the immune system is robust and wound closure is timely, inflammation is generally minimal and self-limiting, reflecting a natural resistance to chronic inflammation and infection; refer to the normal physiological conditions [40].

### 3.8. The Pedunculitis Chronica Deformans (PCD) Is a Consequence of Impaired Wound Healing

When tissue regeneration is impaired and immunosuppression occurs, wound healing disorders can develop. These conditions can trigger an inflammatory response that exceeds physiological levels, resulting in secondary or delayed wound healing, which is typically associated with excessive scar formation [41]. Instead of forming a uniform, continuous layer, the resulting scar tissue is a braided, irregular structure composed of fibrous bundles [42,43]. Such irregular scarring disrupts critical signaling pathways between the wound epidermis and the regenerating blastema [40]. The early chondrocytes, which originated from periosteal antlerogenic stem cells, form blastemas at the outer edges of the wound that develop into chondro-osseous columns during ossification [44,45]. The morphological examination of abnormal pedicles and AABs indicated that these structures originate from sparse, scattered residual osteoblasts and osteo-chondroid trabeculae in the irregular scar tissue.

**Figure 10:**
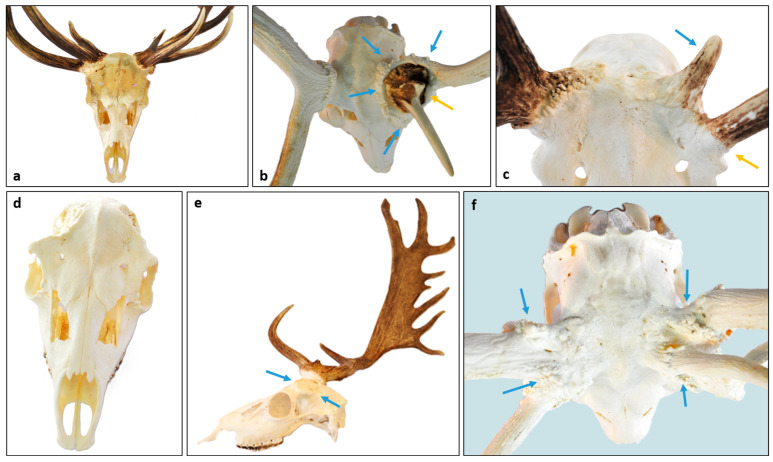
Morphological changes in Pedunculitis Chronica Deformans (PCD) suggest disrupted osteogenesis of the pedicle and antlers by scar tissue. (**a**) Bilateral aberrant antler beams (AAB) can be considered derivatives of antler-forming buds that have retained their developmental capacity between the dissecting scar tissues. They correspond to the phenomenon described as spike-on-one-side (SOOS) but can also be bilateral. (**b**) The top view of a velvet phase trophy shows intramembranous ossification (blue arrows) around the left, but it is not casted AAB (yellow arrow). In this case, the AAB caused the lateralization of ossification originating from the antlerogenic stem cells (ASC) of the chondro-osseous tissue. This process increases the diameter of the pedicle and moves it laterally. (**c**) In cases of severe ASC loss, little residual antler tissue can form (blue arrow), typically on the periphery, often located on the orbital arch (yellow arrow). (**d**) Bilateral total pedicle loss leads to the “hummel” condition. (**e**) In the place of the left pedicle, only a widened bone ridge-like edge remained (blue arrows), which did not correspond to secondary healing after fracture. The widening of the right pedicle is suggestive of PCD, and the long brow tine and massive palm formation can be considered compensatory growth. (**f**) A bony trophy can only indirectly evidence scar formation. However, its appearance with small nodules (blue arrows) corresponds to scars dissecting the chondro-osseous tissue.

**Figure 11:**
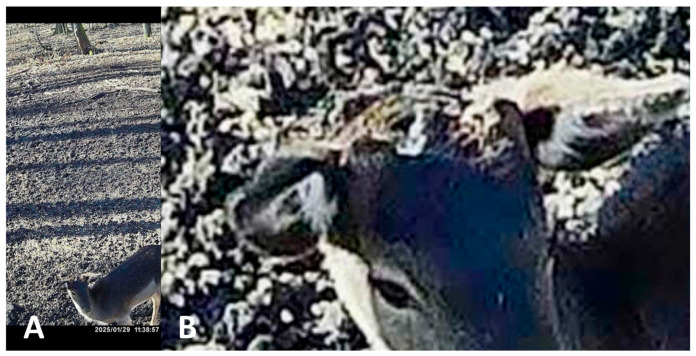
Trail camera footage from around the village of Szakcs (South Hungary, Middle Europe) from our study area. (**A**) This living “hummel” fallow deer (*Dama dama*) buck proves that the loss of antlers on both sides is survivable. (**B**) However, the survival time is questionable. The magnification shows a rather large loss of skull fragments on the left side.

The most obvious manifestation of this is the phenomenon described by Rachow et al., which is the “spike-on-one-side” (SOOS) [6], but it is not exclusively unilateral (Figure 5a,b and Figure 10a,b). In this condition, the pedicle periosteum undergoes appositional intramembranous ossification but widens unevenly due to scarring, often extending laterally and even approaching the orbital ridge (Figure 10c). This abnormal growth pattern results in further shortening and more pronounced bone loss in the pedicle, which can ultimately lead to a “hummel” stage (Figure 10d and Figure 11). The “semi-hummel” condition can also occur: In this case, there is an antler on one side showing minor deviations only, while on the other side it cannot develop (Figure 1 and Figure 10e). In place of the missing pedicle, usually only a widened bone ridge remained, which did not correspond to the morphology of a secondarily healed fracture. These trophies are thought to correspond to cases in which, despite a significant bilateral weight difference, the animals are able to keep their heads upright (Figure 1). In these cases, there is enough time for the development of a compensatory head-hold with neck muscle hypertrophy during the period of gradual weight gain on the intact side, providing an adequate visual field [46]. Sudden weight loss, observed during non-simultaneous shedding, is associated with an oblique head position [47]. Unilateral antler loss due to trauma would cause the same.

The pathomechanism and morphological characteristics of the lesions described do not correspond to any known bone disease, including neoplastic transformations, but show notable similarities to Garré’s sclerosing chronic non-suppurative osteomyelitis. This condition, which typically affects the mandible, is characterized by its “onion-skinning” bone deformation due to mild irritation or bacterial infection resulting from a proliferative periosteal reaction [48]. We have termed this unique condition Pedunculitis Chronica Deformans (PCD). In PCD, the osteoblasts of the peduncular surface ossify intramembranously but fail to form organized, parallel lamellar bone. Instead, they create irregular ridges derived from osseochondroid columns. The top of these ridges retains variable amounts of antlerogenic stem cells. PCD compromises the structural integrity of both the pedicle and the forming antler, occurring independently of any prior injury. The bony trophy can only provide indirect evidence of scarring. However, the appearance of small osseous nodules is consistent with scars that dissect bone tissue (Figure 10f).

**Figure 12:**
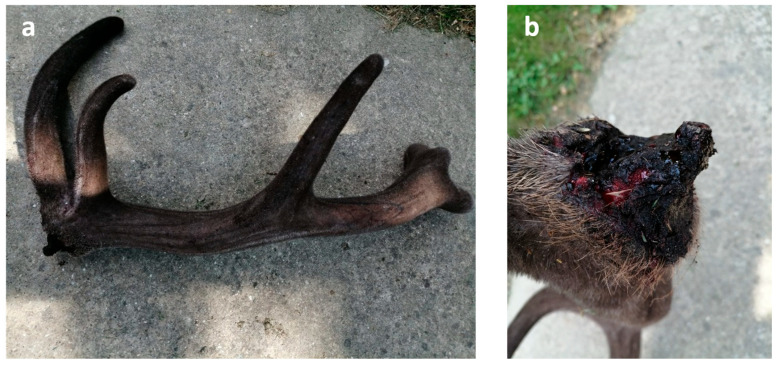
Antlers broken-out with an irregular plane in the velvet phase. (**a**) There is no evidence of a major traumatic force on the antler; therefore, it can be assumed that the pathological fracture was due to the antler’s own weight. (**b**) An irregular fracture surface, which is incompatible with primary external trauma, formed above the casting plane.

Antler casting is primarily dependent on osteoclast activity across the pedicle, with the greatest extent of bone resorption occurring at the casting plane [40]. Under physiological conditions, this process does not result in complete regeneration; instead, the pedicle shortens while its diameter increases through appositional growth [1,40]. In case of healthy casting, the surface of the pedicle remains slightly concave and prickly due to fractured trabeculae, but the pedicle itself retains a circular cross-section and relatively smooth exterior [1]. However, during PCD, there is a notable reduction in both osteoblasts, which are essential for rebuilding the tissues, and also in osteoclasts involved in antler shedding. The distribution of osteoclasts becomes irregular and no longer concentrates exclusively on the casting plane, leading to an uneven casting surface. This disrupted remodeling weakens the pedicle structurally, which can render it incapable of supporting the developing antler, leading to fractures under the antler’s weight even under the velvet phase (Figure 12). PCD is thus a primary contributor to pathological pedicle fractures. Differentiating whether the irregular shedding surface is a result of an atypical, remodeled casting plane or a pathological fracture requires close examination of the fracture surface and careful consideration of the timing relative to the antler shedding period. Previous work by U. Kierdorf and colleagues described an instance where a pathological condition was predisposed to the antler breakage following a velvet-stage injury, which compromised the antler but not the pedicle. They underscored that the underlying pathologic process is necessary in weakening the antler structure, thus predisposing it to fractures [28].

Thus, PCD can be defined as a chronic inflammation primarily affecting the pedicle tissue, caused by casting wound closure or enchondral ossification disturbance of the pedicle. The consequent scarring causes disturbance of pedicle and antler regeneration and weakening of their structure, creating the potential for pathological fracture, superinfection, and the development of fatal intracranial inflammation.

The etiology of PCD remains unknown. Although bacterial infection appears to be a likely cause [9,10,11], the possibility of secondary superinfection cannot be excluded. To the best of our knowledge, the involvement of other infectious agents has not been reported so far. The accumulation of toxic substances in antlers is a well-documented phenomenon [49,50,51]; however, their role in the development of antler or pedicle abnormalities remains unclear.

### 3.9. Peduncular–Dermal Junction (PDJ); Structural and Pathological Insights

**Figure 13:**
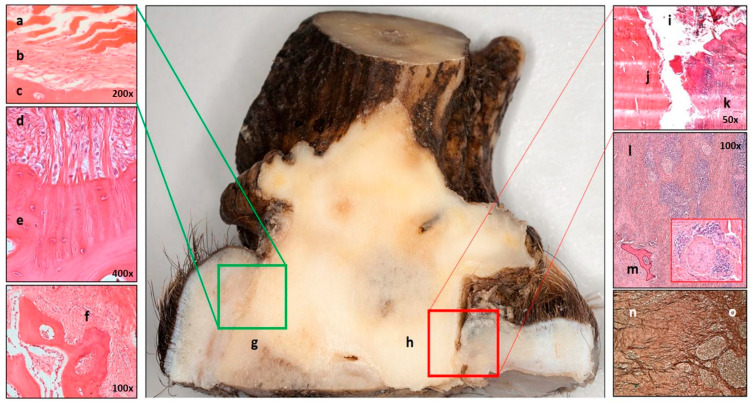
The peduncular–dermal junction (PDJ) and its damage on sagittally sectioned peduncle and peripeduncular soft tissue. The green square indicates healthy, and the red square indicates a diseased area from which excisions were made for microscopic examination. (**a**–**g**) On the left, the histological components of the PDJ are shown. The epidermis (**a**) is directly attached to the bone (**c**) surface without dermis (**b**). (**d**,**e**) Sharpey’s fibres anchor the periosteum (**d**) to the bone (**e**). (**f**) At lower magnification, the bone surface roughness is visible, which increases the adhesion surface. The trabecular bone is on the left side of the picture; the periosteum is on the right. (**h**–**o**) Damage to the PDJ caused periosteal bone discohesion (**h**), allowing the accumulation of foreign, contaminated substances, such as plant fibres, purulent exudate, and tissue debris, in the resulting gap (**i**). Signs of superficial osteomyelitis are a surface covered with devitalized bone particles (**j**) and an accumulation of inflammatory cells in the skin, visible as a bluish discoloration (**k**). In the deeper soft tissue, a massive, predominantly perineural (see insert) and perivascular arrangement of lymphoplasmacytic inflammatory cell infiltration (**l**) with bone sequestrum (**m**) is seen. Chronic inflammation is characterized by fibrosis (**n**), which causes damage to the elastic fiber matrix (**o**) and disruption of Sharpey’s fibers, resulting in further enlargement of the gap with shrinkage of the peripeduncular tissue.

In diseased individuals, we observed a distinct macroscopic separation between the peri-peduncular soft tissues—including the skin, subcutaneous connective tissue, and periosteum—and the underlying bone (Figure 13h). This separation suggests that damage to a critical peripheral structural tight junction around the pedicle may contribute significantly to disease development.

In mammals, the only hitherto characterized bony structure that penetrates the integument is the tooth, whose anatomical structure, especially at the periodontal junction, is well-discussed [52]. This junction isolates the heterogeneous microbial flora of the oral cavity from the rest of the body through the specialized structure of the oral mucosa [52]. Antlers in *Cervidae* are bony structures that penetrate the integument when the velvet is shed and the continuity of the skin is interrupted [40]. This breach in the integument—the body’s first line of defense—potentially exposes the animal to pathogens from the surrounding environment, particularly around the dry antler. We described the soft tissue components around the pedicle as an independent structure referred to as the pedunculo–dermal junction (PDJ), which we believe plays a crucial role in maintaining integumentary integrity. Microscopically, the PDJ is composed of three primary elements: (1) The direct connection of the distal edge of the epidermis without an intervening dermis or subcutaneous layer to the pedicle periosteum [44]; (2) Sharpey’s fibers anchoring the periosteum firmly to the bone [53], and (3) the microscopic roughness of the distal peduncular bone surface, which is augmenting the contact area and potentially enhancing barrier strength (Figure 13a–g). Li et al. demonstrated that the distal third of the pedicle periosteum is more resistant to detachment than the proximal two-thirds [54], supporting our hypothesis that the PDJ is an independent histomorphological structural unit. In other words, segments of peri-peduncular soft tissue of different heights have different significance not only in antler regeneration [55] but also in the closure of the integument after casting of the velvet.

We found that damage to the PDJ caused periosteal-bone discohesion, allowing the accumulation of external contaminants, such as plant fibers in the resulting gap, and introducing pathogens, which bring about superinfections, triggering acute inflammation characterized by purulent exudate and tissue debris. The fibrotic remodeling of the connecting tissue leads to shrinkage of the surrounding elastic fiber matrix and the disruption of Sharpey’s fibers, creating a favorable environment for chronic inflammation around the pedicle (Figure 13h–o). The process can continue for several years, as evidenced by the case we identified in the velvet phase (Figure S2). The pathomechanism bears similarities to parodontitis, where persistent inflammation is exacerbated by compromised structural integrity [52].

## 4. Conclusions

To investigate the antler anomalies in Hungarian Cervidae populations—including fallow deer (*Dama dama*, DD), roe deer (*Capreolus capreolus*, CC), and red deer (*Cervus elaphus*, CE)—we described a chronic inflammatory disease—named Pedunculitis Chronica Deformans (PCD)—of the pedicle. As a result of our work, we have identified morphological characteristics that enable veterinarians, wildlife biologists, and game managers to recognize animals affected by this disease clearly. This paves the way for the collection of accurate epidemiological data and the establishment of homogeneous groups of affected individuals, which in turn will facilitate investigation into the underlying causes and may ultimately contribute to the development of potential treatment strategies. In this study, we introduce a morphological scoring system for this chronic osteomyelitis-like disease. Our work includes a detailed characterization of the peduncular–dermal junction (PDJ) as a distinct anatomical structure, emphasizing its significance as a key factor in the initiation and progression of the disease. This study provides a more comprehensive understanding of these deformities and establishes a foundation for future diagnosis and treatment.

## Figures and Tables

**Table 1:** Detailed description of the RAPS features.

Rose	R1	Lack of continuity in the grain of the rose is longer than 5 mm but less than 1 cm.
	R2	Rose deformity (also visible on cast antlers).
	R3	Loss of rose granularity is greater than 1 cm.
Antler	A1	Supernumerary tine originating from the rose can be a sign of an extra antler bud.
	A2	Visually assessed length asymmetry between the left and right brow tines.
	A2.1	Difference from trophy register control group (TRCG), which is derived from data of the Hungarian Trophy Register after exclusions of cases marked abnormal.
	A3	Visually determined asymmetry in the main beam length between the two sides.
	A3.1	Difference relative to the TRCG.
	A4	Absence of antlers or aberrant antler beams.
Pedicle	P1	Pitting on the proximal surface of the pedicle.
	P2	The pedicle deformity characteristic of PCD in Fallow deer (*Dama dama*) is the deviation from the regular circle diameter more than 4 mm, also visible with the eye.
	P3	Shortening of the pedicle more than physiological.
	P4	Widening of the pedicle beyond the normal extent.
	P5	Peduncle fistula.
	P6	Free casting plane from which does not protrude any antler–bony protuberance.
	P7	More than fifty percent of the casting plane of the pedicle is in or below the plane of the skull, which is considered to be the absence of it.
Skull	S1	The pitting extends on the skull surface.
	S2	Spongy remodelation of skull bones where the cavities are larger than at intertrabecular spaces in spongious bone.
	S3	Discohesion along the interosseous suture.
	S4	Separation of the cortical bones is an independent sign of bone displacement.
	S5	A fistula mouth opens distant from the pedicle on the skull.
	S6	The center of the pedicle has shifted significantly, mostly towards the brow ridge.

**Table 2:** Severity grades of the RAPS features.

Grade I	R1, A1, P1, S1
Grade II	P2, R2, A2, A2.1, P2, P3, P4, S2, S6
Grade III	R3, A3, A3.1, P5, P6, S4
Grade IV	A4, P7, S3, S4

## Data Availability

The original contributions presented in this study are included in the article/Supplementary Material. Further inquiries can be directed to the corresponding authors.

## Supplementary Materials

The following supporting information can be downloaded at: https://www.mdpi.com/article/10.3390/ani15111530/s1, Figure S1: Heatmap of associations among RAPS (rose, R; antler A; pedicle, P; and skull, S) characteristics of the 50 studied fallow deer (*Dama dama*). Figure S2: Peduncular–dermal junction (PDJ) separation during the velvet phase. (a) Left side, the abnormal antler cut into pieces for fixation with its pedicle and surrounding skull roof. (b) Sagittal section of the velvet antler rose and pedicle. The skin–bone discohesion below the burr is visible (arrow). (c) On the other side of this section, the separation between the skin and pedicle is also visible, which is indicative of its extension. (d) The dark discoloration in the gap due to hemorrhage proves that the process occurred in a living animal. Table S1: The incidence of aberrant trophies among fallow deer *(Dama dama*, DD) was notably higher than in roe deer (*Capreolus capreolus*, CC) and red deer (*Cervus elaphus*, CE), with ten times more anomalies in DD than in CC (50 vs. 5) and approximately 16 times more than CE (50 vs. 3). When adjusted for population density, DD appeared to be impacted the most by these deformities: 34 times more frequently than CC and 50 times more than CE, suggesting a unique vulnerability. Table S2: Cervidae trophies collection, five roe deer (*Capreolus capreolus*, CC) trophies exhibited features characteristic of Pedunculitis Chronica Deformans (PCD). Table S3: Evaluation of Pedunculitis Chronica Deformans (PCD) signs on red deer (*Cervus elaphus*, CE). Table S4: Characterization of the Aberrant Trophies with RAPS (rose, R; antler A; pedicle, P; and skull, S) characteristics and their statistical evaluation. Table S5: Analysis of Pedicle Ellipticity among fallow deer (*Dama dama*, DD).

## Abbreviations

The following abbreviations are used in this manuscript:

AAB	aberrant antler beams
ASC	antlerogenic stem cell
B&C	Boone and Crockett Club
CC	*Capreolus capreolus* (roe deer)
CE	*Cervus elaphus* (red deer)
CIC	International Council for Game and Wildlife Conservation
CT	computed tomography
DD	*Dama dama* (fallow deer)
HTR	Hungarian trophy register
MCG	morphology control group
PCD	Pedunculitis Chronica Deformans
PDJ	peduncular–dermal junction
RAPS	rose, antler, pedicle, skull
SOOS	spike-on-one-side
TRCG	trophy register control group

## References

[1] Goss R.J. (1983). Deer Antlers: Regeneration, Function, and Evolution.

[2] Landete-Castillejos T., Currey J., Estevez J., Fierro Y., Calatayud A., Ceacero F., Garcia A., Gallego L. (2010). Do drastic weather effects on diet influence changes in chemical composition, mechanical properties and structure in deer antlers?. Bone.

[3] ‘One in a Million’ Three-Antler Deer Spotted in US-BBC News. https://www.bbc.com/news/world-us-canada-50440102.

[4] Rörig A. (1900). Über Geweihentwickelung und Geweihbildung. Arch. Für Entwicklungsmechanik Org..

[5] Robinette W.L., Jones D.A. (1959). Antler Anomalies of Mule Deer. J. Mammal..

[6] Rachlow J.L., Lee R.M., Riley R.K. (2003). Abnormal Antlers and Pedicles on Rocky Mountain Elk in Northern Arizona. Southwest. Nat..

[7] Karns G.R., Ditchkoff S.S. (2013). Trauma-induced malformed antler development in male white-tailed deer. Wildl. Soc. Bull..

[8] Karns G.R., Ditchkoff S.S. (2012). Antler Breakage Patterns in White-tailed Deer. J. Southeast. Assoc. Fish Wildl. Agencies.

[9] Kierdorf U., Flohr S., Gomez S., Landete-Castillejos T., Kierdorf H. (2013). The structure of pedicle and hard antler bone in the European roe deer (*Capreolus capreolus*): A light microscope and backscattered electron imaging study. J. Anat..

[10] Henshaw J. (1971). Antlers—The Unbrittle Bones of Contention. Nature.

[11] Currey J.D., Landete-Castillejos T., Estevez J., Ceacero F., Olguin A., Garcia A., Gallego L. (2009). The mechanical properties of red deer antler bone when used in fighting. J. Exp. Biol..

[12] Kaufman D.W., Kaufman G.A. (2019). Extreme Antler Malformation in a Mule Deer (*Odocoileus hemionus*) in Northcentral Kansas. Trans. Kans. Acad. Sci..

[13] Faragó S., Köller J., Zoltán A. (2009). Természeti-vadászati örökségünk. A Legkiválóbb Vadásztrófeák.

[14] Nimród-Mi Lesz Veled, Lábodi Dám?-Napjaink Dámgazdálkodási Problémái a SEFAG Zrt. Területén-Nimród-What Will Happen to You, the Fallow Deer of Labod?-The Problems of Fallow Deer Management in the Area of SEFAG LTD. https://nimrod.hu/hirek/milesz.

[15] Gál J., Janosi K., Marosan M., Sugár L. (2011). Purulent inflammation of the basis of antler in fallow deer (*Dama dama*) caused by Staphylococcus xylosus. Magy. Allatorvosok Lapja.

[16] Baumann C.D., Davidson W.R., Roscoe D.E., Beheler-Amass K. (2001). Intracranial abscessation in white-tailed deer of North America. J. Wildl. Dis..

[17] Cohen B.S., Belser E.H., Killmaster C.H., Bowers J.W., Irwin B.J., Yabsley M.J., Miller K.V. (2015). Epizootiology of Cranial Abscess Disease in White-Tailed Deer (*Odocoileus virginianus*) of Georgia, USA. J. Wildl. Dis..

[18] Timmons J., Shaub M., Scherer L., Gereg I., Maxwell L., Potts L., Stevens M., Vile M., Miller E.A., Niedringhaus K.D. (2025). Pathologic Findings of Cranial Abscesses Involving the Pituitary Gland in Free-Ranging White-Tailed Deer (*Odocoileus virginianus*) in Pennsylvania. Animals.

[19] Karns G.R., Lancia R.A., Deperno C.S., Conner M.C., Stoskopf M.K. (2009). Intracranial Abscessation as a Natural Mortality Factor for Adult Male White-Tailed Deer (*Odocoileus virginianus*) in Kent County, Maryland, USA. J. Wildl. Dis..

[20] Hudson R.J., Drew K.R., Baskin L.M. (2010). Wildlife Production Systems: Economic Utilisation of Wild Ungulates.

[21] Behaviour and Management of European Ungulates: Professor Marco Apollonio and Professor Rory Putman: 978-184995-122-7-Whittles Publishing. https://www.whittlespublishing.com/Behaviour_and_Management_of_European_Ungulates.

[22] National Game Management Database. http://www.ova.info.hu/index-en.html.

[23] R Core Team (2023). R: A Language and Environment for Statistical Computing.

[24] Wickham H. (2016). ggplot2: Elegant Graphics for Data Analysis.

[25] Kassambara A. ggpubr: “ggplot2” Based Publication Ready Plots; R Package Version 0.6.0; 2023. https://cran.r-project.org/web/packages/ggpubr/index.html.

[26] Pedersen T.L., RStudio ggraph: An Implementation of Grammar of Graphics for Graphs and Networks; R Package Version 2.2.1; 2024. https://cran.r-project.org/web/packages/ggraph/index.html.

[27] Kolde R. pheatmap: Pretty Heatmaps; R Package Version 1.0.12; 2019. https://cran.r-project.org/web/packages/pheatmap/index.html.

[28] Kierdorf U., Kierdorf H., Konjević D. (2013). Pathological fracture of a red deer antler secondary to purulent inflammation—A case report. Vet. Arh..

[29] Davidson W.R., Nettles V.F., Hayes L.E., Howerth E.W., Couvillion C.E. (1990). Epidemiologic features of an intracranial abscessation/suppurative meningoencephalitis complex in white-tailed deer. J. Wildl. Dis..

[30] Cierny G., Mader J.T., Penninck J.J. (2003). A clinical staging system for adult osteomyelitis. Clin. Orthop..

[31] Tiemann A., Hofmann G.O., Krukemeyer M.G., Krenn V., Langwald S. (2014). Histopathological Osteomyelitis Evaluation Score (HOES)—An innovative approach to histopathological diagnostics and scoring of osteomyelitis. GMS Interdiscip. Plast. Reconstr. Surg. DGPW.

[32] Shed Antlers: Different Pedicle Types|Every Spring Deer Shed Their Antlers and Many Avid Sportsmen Take to the Woods to Find Sheds. Here We Take an In-Depth Look at the Different Shapes of...|By MSU Deer LabFacebook|Facebook. https://www.facebook.com/msu.deerlab/videos/shed-antlers-different-pedicle-types/188785065871222/.

[33] Bubenik A.B., Bubenik G.A., Bubenik A.B. (1990). Epigenetical, Morphological, Physiological, and Behavioral Aspects of Evolution of Horns, Pronghorns, and Antlers. Horns, Pronghorns, and Antlers: Evolution, Morphology, Physiology, and Social Significance.

[34] Kierdorf U., Stoffels E., Stoffels D., Kierdorf H., Szuwart T., Clemen G. (2003). Histological studies of bone formation during pedicle restoration and early antler regeneration in roe deer and fallow deer. Anat. Rec. Part A Discov. Mol. Cell. Evol. Biol..

[35] Li C., Yang F., Sheppard A. (2009). Adult stem cells and mammalian epimorphic regeneration-insights from studying annual renewal of deer antlers. Curr. Stem Cell Res. Ther..

[36] Sybenga A.B., Jupiter D.C., Speights V.O., Rao A. (2020). Diagnosing Osteomyelitis: A Histology Guide for Pathologists. J. Foot Ankle Surg..

[37] Li C. (2021). Residual antler periosteum holds the potential to partially regenerate lost antler tissue. J. Exp. Zool. Part A Ecol. Integr. Physiol..

[38] Li C., Suttie J.M., Clark D.E. (2004). Morphological observation of antler regeneration in red deer (*Cervus elaphus*). J. Morphol..

[39] Agriculture and Natural Resources Marketing. Deer Ecology & Management Lab. https://www.msudeer.msstate.edu/.

[40] Kierdorf U., Kierdorf H. (2011). Deer antlers—A model of mammalian appendage regeneration: An extensive review. Gerontology.

[41] Stadelmann W.K., Digenis A.G., Tobin G.R. (1998). Physiology and healing dynamics of chronic cutaneous wounds. Am. J. Surg..

[42] Jonathan Sherratt’s Research: Scar Formation. https://www.macs.hw.ac.uk/~jas/researchinterests/scartissueformation.html.

[43] Cumming B.D., McElwain D.L.S., Upton Z. (2009). A mathematical model of wound healing and subsequent scarring. J. R. Soc. Interface.

[44] Li C., Suttie J.M., Clark D.E. (2005). Histological examination of antler regeneration in red deer (*Cervus elaphus*). Anat. Rec. Part A Discov. Mol. Cell. Evol. Biol..

[45] Landete-Castillejos T., Kierdorf H., Gomez S., Luna S., García A., Cappelli J., Pérez-Serrano M., Pérez-Barbería J., Gallego L., Kierdorf U. (2019). Antlers-Evolution, development, structure, composition, and biomechanics of an outstanding type of bone. Bone.

[46] Newman B.A., D’Angelo G.J. (2024). A Review of Cervidae Visual Ecology. Animals.

[47] Széchenyi Z. A Szarvas Selejtezése (Selective Culling of Red Deer). Régi Vadászkönyvek. https://www.vadaszkonyv.com/szechenyi-zsigmond-a-szarvas-selejtezese/.

[48] Pathology Outlines-Osteomyelitis Overview. https://www.pathologyoutlines.com/topic/mandiblemaxillaosteomyelitis.html.

[49] Kierdorf U., Kierdorf H. (2006). Roe and red deer antlers as bioindicators of pollution of deer. Vet Arh..

[50] Ludolphy C., Kierdorf U., Kierdorf H. (2021). Lead concentrations in antlers of European roe deer (*Capreolus capreolus*) from an agricultural area in Northern Germany over a 119-year period—A historical biomonitoring study. Environ. Sci. Pollut. Res..

[51] Lakatos I., Babarczi B., Molnár Z., Tóth A., Skoda G., Horváth G.F., Horváth A., Tóth D., Sükösd F., Szemethy L. (2024). First Results on the Presence of Mycotoxins in the Liver of Pregnant Fallow Deer (*Dama dama*) Hinds and Fetuses. Animals.

[52] Nanci A., Bosshardt D.D. (2006). Structure of periodontal tissues in health and disease. Periodontology 2000.

[53] Harris H.A. (1928). Bone Formation and the Osteoblast. Lancet.

[54] Li C., Suttie J.M. (2003). Tissue collection methods for antler research. Eur. J. Morphol..

[55] Li C., Yang F., Li G., Gao X., Xing X., Wei H., Deng X., Clark D.E. (2007). Antler regeneration: A dependent process of stem tissue primed via interaction with its enveloping skin. J. Exp. Zool. Part A Ecol. Genet. Physiol..

