# Supplementary material for: When Antlers Grow Abnormally: A Hidden Disease Behind Common Cervid Trophy Deformities, Introducing Pedunculitis Chronica Deformans
Source: Animals (Basel). 2025 May 23;15(11):1530. doi: 10.3390/ani15111530 (PMC12153838; doi:10.3390/ani15111530)
Supplement: Supplementary file 1 [file animals-15-01530-s001.zip › Figure S1.pdf]

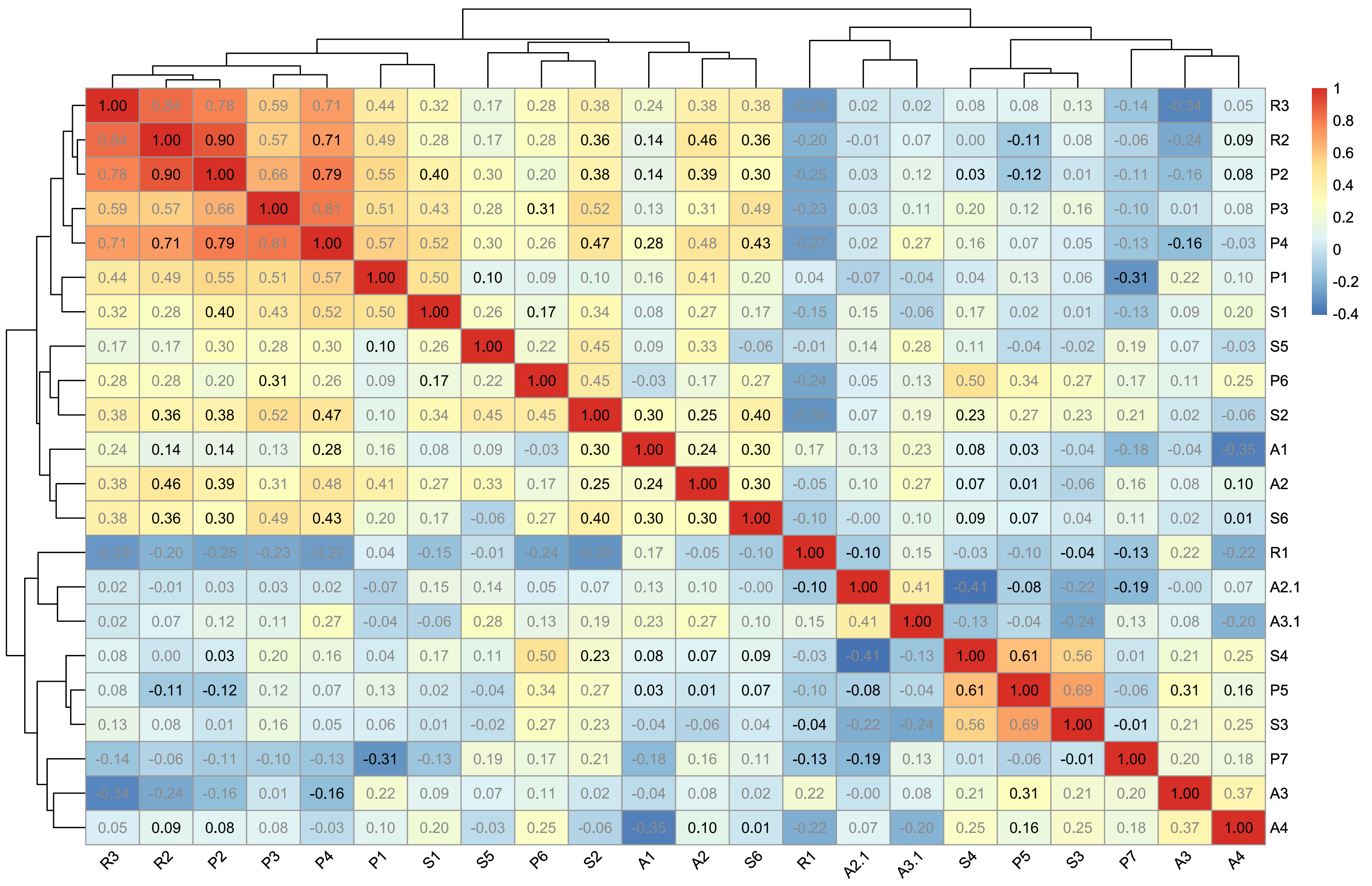

Supplementary Figure S1. Association among RAPS (Rose, R; Antler A; Pedicle, P; and Skull, S) characteristics of the 50 studied fallow deer (*Dama dama*). For this figure and for Fig. 5d, RAPS data- normally scored on each side- were aggregated giving an individual a score of 2 if the given symptom was present on both sides (where applicable) and a score of 1 if it was only present on one side. Numbers indicate Pearson correlation coefficients. Black numbers indicate significant correlations.
