# Supplementary material for: When Antlers Grow Abnormally: A Hidden Disease Behind Common Cervid Trophy Deformities, Introducing Pedunculitis Chronica Deformans
Source: Animals (Basel). 2025 May 23;15(11):1530. doi: 10.3390/ani15111530 (PMC12153838; doi:10.3390/ani15111530)
Supplement: Supplementary file 1 [file animals-15-01530-s001.zip › Figure S2.pdf]

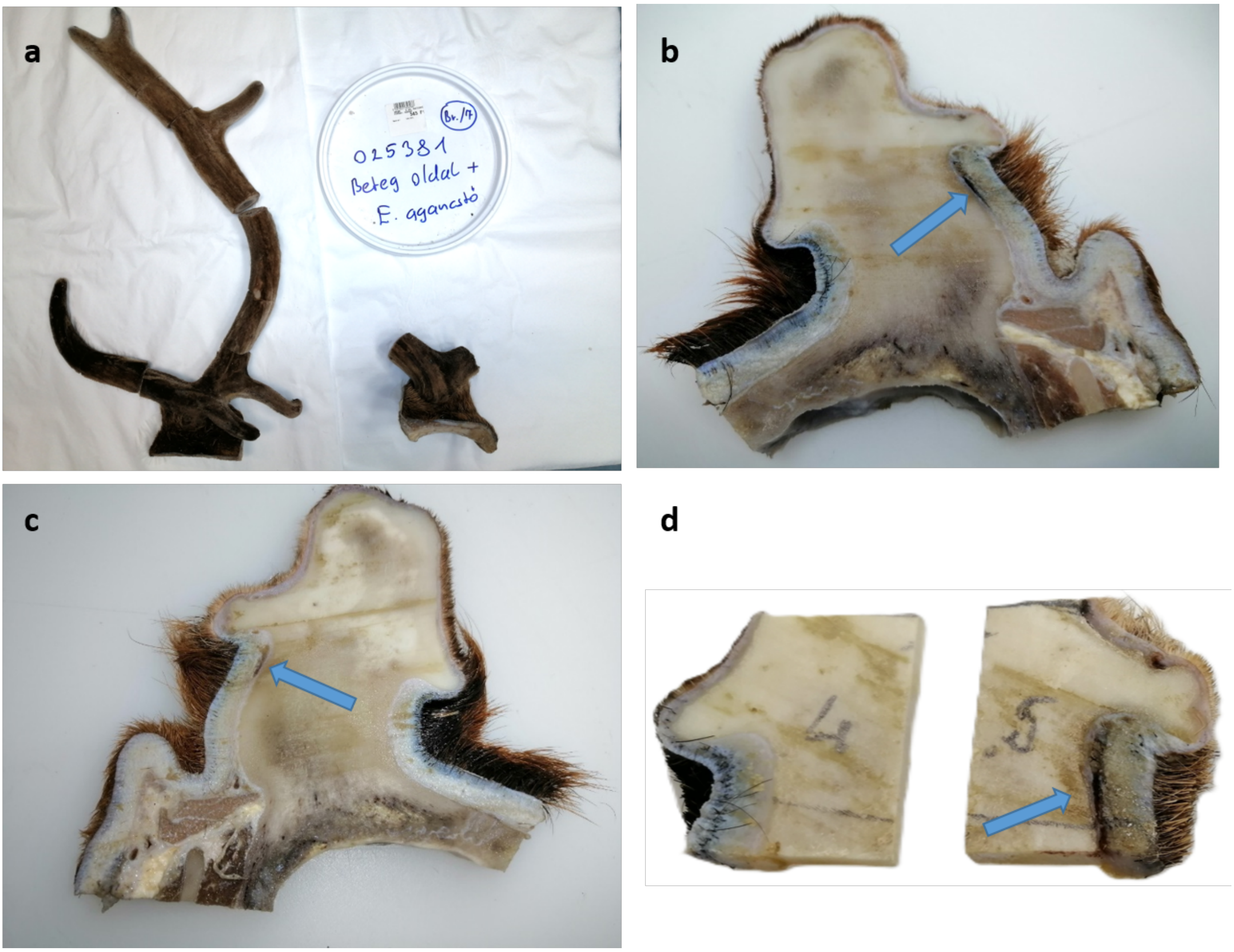

Supplementary Figure S2. Peduncular-Dermal Junction (PDJ) separation during the velvet phase. **a** Left side, the abnormal antler cut into pieces for fixation with its pedicle and surrounding skull roof. **b** Sagittal section of the velvet antler rose and pedicle. The skin-bone discohesion below the burr is visible (arrow). **c** On the other side of this section, the separation between the skin and pedicle is also visible, which is indicative of its extension. **d** The dark discoloration in the gap due to hemorrhage proves that the process occurred in a living animal.
